# ATP Binding Cassette Transporter ABCA7 Regulates NKT Cell Development and Function by Controlling CD1d Expression and Lipid Raft Content

**DOI:** 10.1038/srep40273

**Published:** 2017-01-16

**Authors:** Heba N. Nowyhed, Shilpi Chandra, William Kiosses, Paola Marcovecchio, Farah Andary, Meng Zhao, Michael L. Fitzgerald, Mitchell Kronenberg, Catherine C. Hedrick

**Affiliations:** 1 La Jolla Institute for Allergy and Immunology, La Jolla, CA, USA; 2 Lipid Metabolism Unit and Center for Computational and Integrative Biology, Massachusetts General Hospital, Harvard Medical School, Boston, MA, USA.

## Abstract

ABCA7 is an ABC transporter expressed on the plasma membrane, and actively exports phospholipid complexes from the cytoplasmic to the exocytoplasmic leaflet of membranes. Invariant NKT (*i*NKT) cells are a subpopulation of T lymphocytes that recognize glycolipid antigens in the context of CD1d-mediated antigen presentation. In this study, we demonstrate that ABCA7 regulates the development of NKT cells in a cell-extrinsic manner. We found that in *Abca7*^−/−^ mice there is reduced expression of CD1d accompanied by an alteration in lipid raft content on the plasma membrane of thymocytes and antigen presenting cells. Together, these alterations caused by absence of ABCA7 negatively affect NKT cell development and function.

Adenosine triphosphate binding cassette (ABC) transporters constitute a group of evolutionary highly conserved cellular transmembrane transport proteins. ABCA1, ABCA7, and ABCA4 are members of the ABCA subfamily and share extensive sequence and structural similarity^1^. Several studies have shown that ABCA proteins are involved in lipid transport^2^,^3^,^4^,^5^. ABCA7 is a full-size, single subunit ABC-transporter consisting of 12 transmembrane-spanning domains^1^,^6^,^7^. Within cells, it is expressed predominantly on the plasma membrane, but it is also detected in intracellular membranes^6^,^7^. ABCA7 is preferentially expressed in the thymus, spleen and fetal liver in both humans and mice, and when exogenously transfected and expressed, can mediate apolipoprotein-derived HDL efflux, similarly to ABCA1^8^. Quazi *et al*. described that ABCA7 actively exported phosphatidylserine from the cytoplasmic to the exocytoplasmic leaflet of membranes^9^. ABCA7 is thought to play an important role in lipid homeostasis in cells of the immune system^10^,^11^. More recently, ABCA7 was found to be significantly associated with phagocytosis in macrophages both *in vivo* and *in vitro*^12^,^13^.

NKT cells are a distinct subset of T lymphocytes that express a single invariant T cell receptor α (TCR) chain encoded by Vα14-Jα18 in mice and Vα24-Jα18 in humans, along with a restricted group of TCRβ chains^14^. NKT cells arise in the thymus from uncommitted CD4^−^CD8^−^ double-negative (DN) precursors. Progression of these cells to the CD4^+^CD8^+^ double-positive (DP) stage occurs in parallel with the random rearrangement of their TCR. Thymocytes expressing a TCR that interacts with CD1d bound to certain self-glycolipids presented by other DP thymocytes enter the NKT cell lineage^15^. After this initial selection event, NKT cell precursors undergo a series of differentiation steps characterized by extensive proliferation and accumulation in the thymus and the sequential expression of several cell surface markers such as CD24, CD44 and NK1.1. Based on surface marker and transcription factor expression, as well as cytokine production, multiple NKT cell subsets have been identified that play important modulatory roles in controlling immune responses to pathogens and disease^16^,^17^.

CD1d is present on DP thymocytes, hepatocytes, macrophages, dendritic cells and some B cells. NKT cell activation is unique in that it is mediated by TCR engagement of lipid antigens presented by CD1d, an MHC class I-like antigen presenting molecule^18^. Upon activation by antigens presented by CD1d, peripheral NKT cells release large quantities of IFNγ and IL-4^19^,^20^. The sources of these antigens include various microbes and also self-antigens^15^. The nature of these autologous lipids (or self-antigens) involved in NKT cell positive selection or in the peripheral activation of NKT cells remains unclear^21^. Several cellular glycosphingolipids, endogenous α-glycosylceramides, lysophosphatidylcholine (LPC), lysoplasmalogens and phosphatidylinositol membrane lipid anchors have been proposed as possible major self-ligands for selecting NKT cells in the thymus and/or activating them in the periphery^22^,^23^,^24^.

CD1d has been localized in lipid raft microdomains^25^,^26^,^27^. These plasma membrane platforms mediate cell signaling and immune responses by concentrating different signaling molecules together. Lipid rafts are composed of cholesterol and glycolipids, and can be identified by cholera-toxin-B and/or caveolin-1 labeling^28^. The inhibition of raft integrity preferentially prevents CD1d antigen presentation^25^,^26^. We previously reported an essential role of another ABC transporter, ABCG1, in NKT cell development and function^29^. ABCG1 regulates intracellular cholesterol homeostasis^30^,^31^, and plays important roles in lymphocyte activation^32^,^33^. We reported that the absence of ABCG1 profoundly reduced thymic NKT cell development and function in a cell-intrinsic manner^34^. Because of our findings on ABCG1, and the role of ABCA7 in lipid transport, here we set out to understand the possible role of another ABC transporter, ABCA7, in NKT cell development and function. We found that ABCA7 also has a major influence on NKT cell function, but the mechanism whereby ABCA7 does this is very different from ABCG1.

## Results

### ABCA7 regulates NKT cell development and frequency

**Figure 1:**
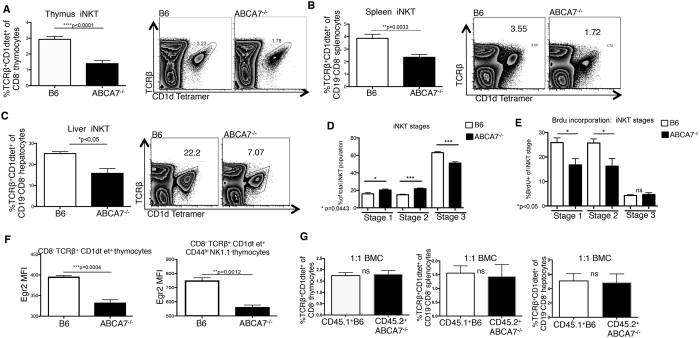
Impaired NKT development in the absence of ABCA7. Frequency of NKT cells (CD8^−^TCRb^+^CD1d-tet^+^) in total (**A**) thymocytes, (**B**) splenocytes, and (**C**) hepatocytes in WT (B6) control versus *Abca7*^−/−^ mice determined by flow cytometry. Representative plots shown for each; graphs represent compiled data from two separate experiments with at least four age- and sex-matched mice per group. (**D**) Frequency of NKT cells at each stage of development in thymus (Stage 1 CD44^lo^NK1.1^−^, Stage 2 CD44^hi^NK1.1^−^, Stage 3 CD44^hi^NK1.1^+^) in WT versus *Abca7*^−/−^ mice. (**E**) Frequency of BrdU^+^ NKT cells at Stages 1-3 of development in thymus analyzed by flow cytometry. (**F**) Mean Fluorescence intensity (MFI) of Egr2 in total NKT cells and in NKT cells at Stage 2 of development within the thymus as calculated by flow cytometry. (**G**) 1:1 mixed bone marrow chimeras (1:1 BMC) were generated by transferring equal numbers of CD45.1 WT control bone marrow and CD45.2 *Abca7*^−/−^ bone marrow cells into CD45.1.2 irradiated hosts. NKT cell development was analyzed by flow cytometry at 12 weeks after reconstitution. Data shown are for thymocytes, splenocytes and hepatocytes, respectively. All data in Fig. 1 are representative of at least two separate experiments with at least four age- and sex-matched mice per group. Data are expressed as mean ± SEM. *p < 0.05, **p < 0.005, ***p < 0.0005, ****p < 0.00005, or ns: not significant, by unpaired, two-tailed Student’s t-test.

We set out to decipher the role that ABCA7 plays in NKT cell development through the evaluation of thymocytes in ABCA7-deficient mice. In *Abca7*^−/−^ mice, we found a 2-fold decrease in frequencies (Fig. 1A) and total numbers (Supplemental Fig. 1) of NKT cells in the thymus, while thymus cellularity, and the frequency of conventional CD4^+^ and CD8^+^ T cells were normal (Supplemental Fig. 1A). Similar reductions in peripheral NKT cells were observed in spleen and liver (Fig. 1B,C). After positive selection in the thymus, NKT cells proliferate and mature in defined stages marked by surface expression of CD44 and NK1.1. Cells with a stage 1 phenotype (CD44^low^ NK1.1^−^) are followed by stage 2 cells, which have increased CD44 expression (CD44^high^ NK1.1^−^). Stage 1 and stage 2 NKT cells are highly proliferative. The upregulation of NK1.1 expression by NKT cells marks stage 3 (CD44^high^ NK1.1^+^) cells, which are mature but less proliferative. We next analyzed thymic NKT cells to determine at what stage NKT cell development was impaired in ABCA7-deficient mice. We found that the numbers of *Abca7*^−/−^ NKT cells were reduced at stages 2 and 3 of development (Supplemental Fig. 1B). Although the numbers were decreased, the frequencies of stage 1 and stage 2 *Abca7*^−/−^ NKT cells were increased (Fig. 1D), leading us to question whether the ABCA7-deficient NKT cells were “stalled” at these early stages of development; thus, unable to progress to the next developmental stage.

To determine whether the reduced number of NKT cells in the absence of ABCA7 was due to a proliferative defect, we measured proliferation of thymic NKT cells at different maturation stages *in vivo* in *Abca7*^−/−^ and C57BL/6 J (WT) control mice by BrdU incorporation (Fig. 1E, and Supplemental Fig. 1C). In *Abca7*^−/−^ mice, the frequencies of Brdu^+^ NKT cells at stages 1 and 2 were significantly reduced compared with NKT cells from WT mice (Fig. 1E). However, the percentages of apoptotic NKT cells (as measured by annexin V^+^ live cells) at stages 1–3 in *Abca7*^−/−^ and WT mice were not significantly different (Supplemental Fig. 1D). These results indicate that the reduced frequency of NKT cells in the thymus in the absence of ABCA7 appears to be due, at least in part, to reduced proliferation, particularly during the early stages of NKT cell development.

In order for a developing NKT cell to proliferate and move to the next stage of development in the thymus, the invariant TCR must interact with a CD1d-expressing DP thymocyte and receive a positive signal. The TCR induced transcription factor Egr2 is highly expressed in precursors of NKT cells undergoing positive selection^35^. Therefore we measured Egr2 in developing NKT cells in both *Abca7*^−/−^ and WT thymi and found a significant reduction of Egr2 expression in *Abca7*^−/−^ NKT cells (Fig. 1F). Strong TCR signals induce Egr2, and therefore we investigated whether deficiency of ABCA7 in NKT cells would affect their TCR-driven activation. We stimulated negatively-enriched NKT cells from thymi of WT and *Abca7*^−/−^ mice with plate bound anti-CD3 and soluble costimulatory anti-CD28 antibody *in vitro* and measured IL-4 and IFNγ production by thymic NKT cells (Supplemental Fig. 1E). We found that *Abca7*^−/−^ NKT cells had normal IL-4 and IFNγ production, suggesting that direct activation through the TCR was normal. Thus, we hypothesized that the defect in NKT cell development occurring in *Abca7*^−/−^ mice is likely a cell-extrinsic effect, possibly resulting from impaired actions of the selecting CD1d^+^ DP thymocytes.

To address a possible cell-extrinsic cause for changes in NKT cell development in *Abca7*^−/−^ mice, we analyzed mixed bone marrow chimeric mice. Irradiated *Rag1*^−/−^ mice were reconstituted with both CD45.1^+^ WT and CD45.2^+^ *Abca7*^−/−^ bone marrow, mixed at a 1:1 ratio, and analyzed 12 weeks after reconstitution. In the presence of wild-type WT thymocytes, ABCA7-deficient NKT cells developed at a normal frequency (Fig. 1G). These results clearly point to a cell-extrinsic mechanism contributing to the impaired NKT cell development in *Abca7*^−/−^ mice. As our data in Fig. 1E,F showing reduced proliferation and reduced Egr2 expression, these data suggested a defect in TCR-CD1d signaling due to a possible defect in CD1d-mediated antigen presentation by DP thymocytes, the cell type responsible for NKT cell positive selection.

### ABCA7 regulates CD1d expression

**Figure 2:**
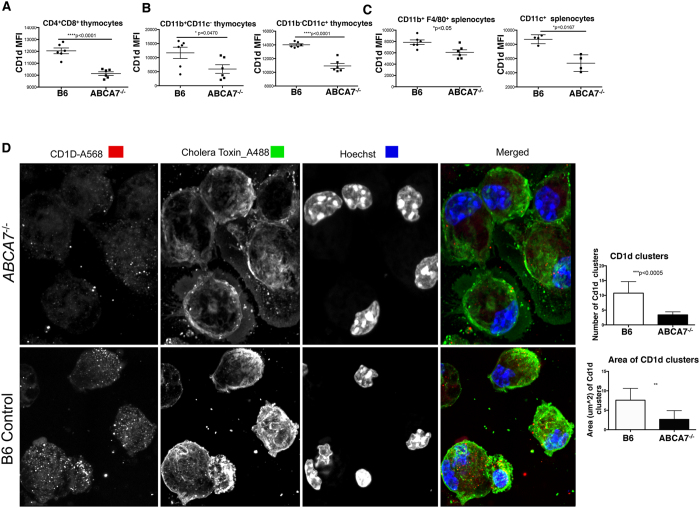
ABCA7 regulates CD1d surface expression and localization in lipid rafts. (**A**) Flow cytometry analysis of CD1d mean fluorescence intensity (MFI) on CD4^+^CD8^+^ (double positive) thymocytes, (**B**) CD11b^+^CD11c^−^ and CD11b^−^CD11c^+^ thymocytes from WT (B6) and *Abca7*^−/−^ mice. (**C**) Flow cytometry analysis of CD1d MFI on CD11b^+^F4/80^+^, and CD11c^+^CD11b^−^ antigen-presenting cells in spleens from WT and *Abca7*^−/−^ mice. Data in Panels A–C are representative of at least 2 separate experiments with at least three age- and sex-matched mice per group. Data are expressed as mean ± SEM. *p < 0.05, ****p < 0.0001, or ns: not significant, by unpaired, two-tailed Student’s t-test. (**D**) Representative images of CD1d and lipid raft colocalization as measured by Cholera toxin-B staining by confocal microscopy on peritoneal macrophages harvested from WT and *Abca7*^−/−^ mice. Hoechst is shown for nuclei staining. Number of CD1d clusters quantified from images from 200 cells (top bar graph). Area of CD1d clusters quantified from images of 200 cells (bottom bar graph). Data are expressed as mean ± SEM. **p < 0.005, ***p < 0.0005, by unpaired, two-tailed Student’s t-test.

Because NKT cells are positively selected by DP thymocytes, we examined whether DP thymocytes from *Abca7*^−/−^ mice had lower CD1d expression. If so, this would be a likely explanation for the observed decrease in NKT cell frequency and number. NKT negative selection is also mediated in part by dendritic cells^36^. We found a significant decrease in surface CD1d expression on DP thymocytes in *Abca7*^−/−^ mice (Fig. 2A). CD1d also was decreased on thymic antigen-presenting cells, including CD11c^+^CD11b^−^ thymocytes and CD11b^+^CD11c^−^ thymocytes (Fig. 2B), cells that may also play a role in negative selection of NKT cells. MHC class II molecules and members of the CD1 family of antigen presenting molecules both traffic via endosomal compartments to load with antigens, and both MHCII and CD1d can be detected within the same endosomal compartments. To determine whether the observed defect was selective to CD1d, or whether it impacted MHCI and MHCII, we measured expression of MHCI and MHCII in thymus. We found that MHCII and MHCI surface expression were normal on thymocytes in the absence of ABCA7 (Supplemental Fig. 1F), verifying that this defect was specific for CD1d and was not a general trafficking defect.

Peripheral NKT cell activation is dependent in part on CD1d-restricted antigen presentation by CD11c^+^ dendritic cells and/or CD11b^+^ F4/80^+^ macrophages. We analyzed CD1d expression on peripheral antigen presenting cells in spleens of *Abca7*^−/−^ mice and found a similar reduction in surface CD1d expression (Fig. 2C). We further studied CD1d expression and localization on peritoneal macrophages isolated from WT and *Abca7*^−/−^ mice (Fig. 2D) using confocal microscopy. On average, CD1d was expressed at a 50% lower density on the surface of ABCA7-deficient macrophages. We also found that the average area of each CD1d cluster was significantly lower in the absence of ABCA7 (Fig. 2D, right graph) suggesting a defect in the aggregation of CD1d on the surface of the cells.

**Figure 3:**
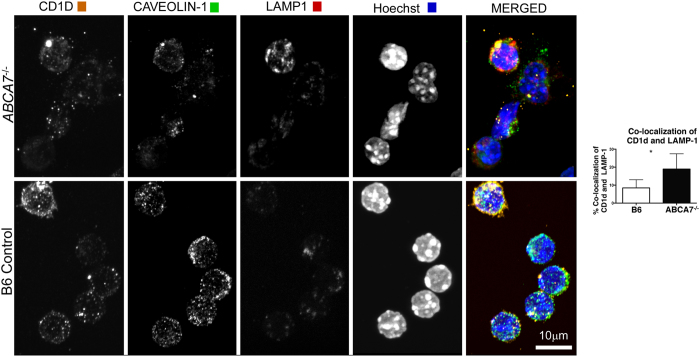
ABCA7 deficiency blocks CD1d trafficking. Co-localization by confocal microscopy of CD1d, Caveolin-1, and LAMP-1 on peritoneal macrophages harvested from WT and *Abca7*^−/−^ mice (representative images). Frequency of co-localization of CD1d with LAMP-1 quantified from images from 200 cells (bar graph). Data are expressed as mean ± SEM. *p < 0.05, by unpaired, two-tailed Student’s t-test.

Using surface anti-CD1d labeling, various groups have shown that internalization of CD1d from the plasma membrane to LAMP-1^+^ intracellular compartments is the underlying mechanism by which CD1d is accumulated in late endosomes and lysosomes^22^,^37^. CD1d molecules are prominently expressed in LAMP-1 lysosomes, and redistribution from lysosomes to the plasma membrane occurs upon lipid antigen loading onto CD1d^38^,^39^. In the absence of efficient antigen loading, CD1d becomes “trapped” and accumulates within late endosomal compartments. Based on these previous findings, we investigated if ABCA7 functions to regulated CD1d trafficking out of LAMP-1^+^ late endosomal compartments to the cell surface. We analyzed co-localization of CD1d and LAMP-1 in WT and *Abca7*^−/−^ peritoneal macrophages by confocal microscopy (Fig. 3). We found a significant increase in co-localization of CD1d and LAMP-1 in the absence of ABCA7. Additional high-resolution images of this co-localization are shown in Supplemental Fig. 2. The observed intracellular accumulation of CD1d within late endosomes suggests that ABCA7 regulates CD1d trafficking out of these intracellular compartments to the cell surface, possibly through the loading of lipid antigen onto CD1d.

The influence of ABCA7 on the trafficking of CD1d out of late endosomal compartments to the cell surface could be related to association of these two molecules. We labeled DP thymocytes with fluorescent antibodies against ABCA7 and CD1d and analyzed the possible association of the two proteins through confocal microscopy (Supplemental Fig. 3A). Based on the overlapping signal from ABCA7 and CD1d, both proteins appear to be localized in close proximity within the same compartment.

### ABCA7 influences lipid raft content

Plasma membranes possess distinct cholesterol- and sphingolipid-rich lipid raft microdomains, which constitute critical sites for signal transduction through various immune cell receptors. Lipid rafts are abundant in the plasma membrane but also in late secretory pathway and endocytic compartments. The dynamic function of lipid rafts to mobilize, aggregate, and crosslink surface receptors has been described as a crucial event in efficient signaling^40^. CD1d is constitutively present within plasma membrane lipid rafts on antigen presenting cells, and this restricted localization is critically important for efficient antigen receptor-mediated activation of NKT cells^27^. Impaired lipid raft distribution is critical for proper CD1d function in NKT cell activation^41^.

**Figure 4:**
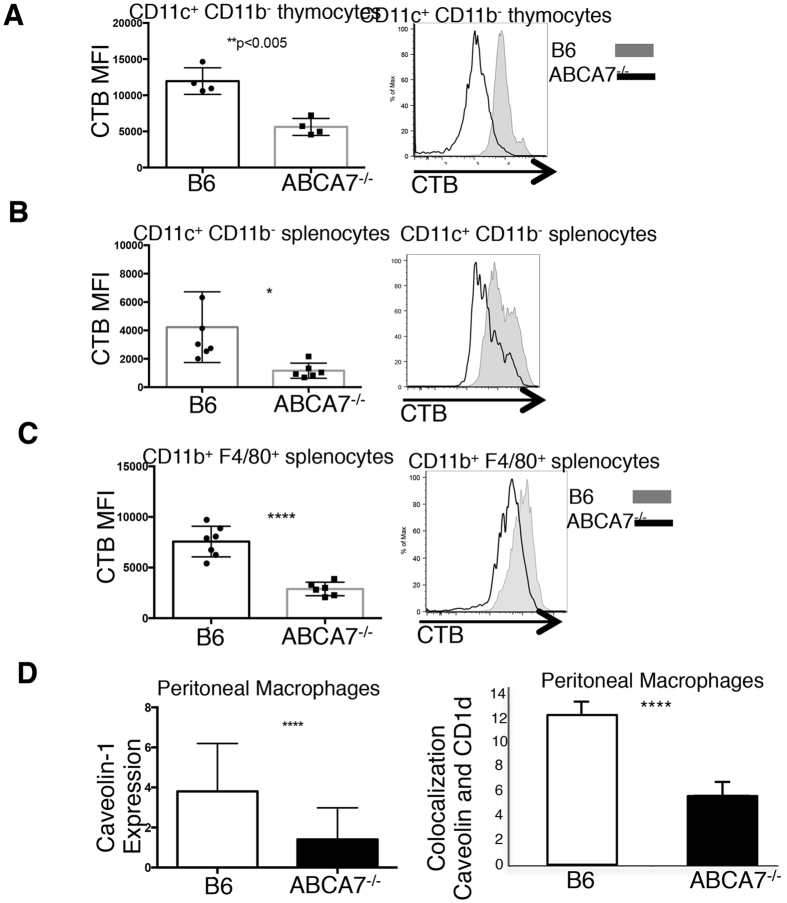
ABCA7 deficiency disrupts lipid rafts in antigen-presenting cells. Flow cytometry analysis of the MFI for Cholera toxin-B staining of lipid rafts on the surface of (**A**) CD11c^+^CD11b^−^ thymocytes, (**B**) CD11c^+^CD11b^−^ antigen-presenting cells from spleen, and (**C**) CD11b^+^F4/80^+^ antigen-presenting cells from spleen in WT (B6) and *Abca7*^−/−^ mice. (**D**) Caveolin-1 expression measured by analysis of confocal microscopy images of 200 peritoneal macrophages isolated from WT and *Abca7*^−/−^ mice (left graph). Frequency of co-localization of Caveolin-1 and CD1d measured by confocal microscopy analysis of 200 peritoneal macrophages isolated from WT and *Abca7*^−/−^ mice (right graph). Data are representative of at least 3 separate experiments with at least three age and sex matched mice per group. P value, unpaired, two-tailed Student’s t-test. *p < 0.05, **p < 0.005, ****p < 0.0005.

There is published evidence that ABCA7 in part functions to traffic lipid complexes from cytoplasmic facing to facing the extracellular milieu^10^. Furthermore, studies have indicated that ABCA7 transports phospholipids from the inner to the outer leaflet of the plasma membrane^3^,^9^. We analyzed lipid rafts by cholera toxin B (CTB) staining on ABCA7-deficient and wild-type APCs in the thymus and the periphery. We found a significant reduction in the number of plasma membrane rafts in the absence of ABCA7 on CD11c^+^CD11b^−^ thymocytes (Fig. 4A), CD11c^+^CD11b^−^ splenocytes (Fig. 4B), and CD11b^+^F4/80^+^ splenocytes (Fig. 4C). We verified these results by analyzing caveolin-1 on peritoneal macrophages harvested from WT and *Abca7*^−/−^ mice through confocal microscopy (Fig. 4D, left graph). Caveolin-1 was significantly reduced in *Abca7*^−/−^ macrophages, verifying a reduction in lipid raft content. Analysis of CD1d co-localization within lipid rafts in WT and *Abca7*^−/−^ cells revealed a significant decrease in CD1d co-localization in ABCA7-deficient macrophages (Fig. 4D, right graph). The reduced area of CD1d clusters observed in *Abca7*^−/−^macrophages is likely due to this defect in lipid rafts and co-localization of CD1d within the lipid rafts. We analyzed CD1d surface expression on *Abca7*^−/−^ thymocytes in direct comparison to *CD1d*^*+*/*−*^ thymocytes and found very similar CD1d expression (Supplemental Fig. 3B). Recent studies have shown that CD1d clustering on antigen-presenting cells is critical for NKT activation^42^. ABCA7 deficiency is critical for our observed NKT phenotype, as diminished expression of surface CD1d alone does not result in reduced NKT cell development, as is observed in *CD1d*^*+*/*−*^ mice, which exhibit close to a 50% reduction in CD1d expression on DP thymocytes, yet have normal NKT cell development^43^. Therefore, our data indicate that ABCA7 plays a multifactorial role in both regulating trafficking of CD1d to the surface, and transporting of lipid, resulting in alterations in lipid rafts as well as reduced co-localization of CD1d within the lipid rafts.

### ABCA7 deficiency in antigen presenting cells results in diminished NKT cell activation

**Figure 5:**
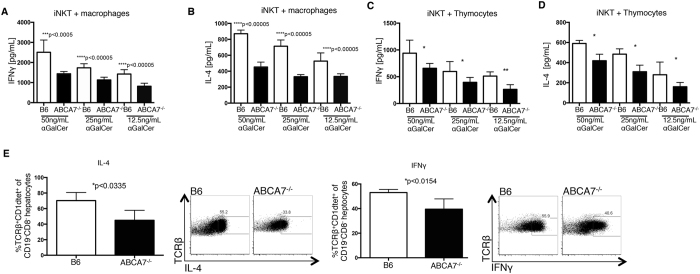
NKT cells display impaired cytokine responses in the absence of ABCA7. Peritoneal macrophages were isolated from WT (B6) and *Abca7*^−/−^ mice, loaded with titrated concentrations of αGalCer for 6 h, and then co-cultured with the primary mouse NKT cell line overnight. Secreted IFNγ (**A**) and IL-4 (**B**) were measured by ELISA. (**C**) Thymocytes were isolated from WT and *Abca7*^−/−^ mice, loaded with titrated concentrations of αGalCer for 6 h, and then co-cultured with a primary mouse NKT cell line overnight. Secreted IFNγ and IL-4 were measured by ELISA. (**E**) Mice were injected intraperitoneally with αGalCer. Livers were collected at 2 h and NKT cells analyzed by intracellular staining for IFNγ and IL-4 production *in vivo*. Representative flow cytometry plots are shown. Graphs represent compiled data. Data are representative of at least two separate experiments with at least three age and sex matched mice per group. P-value, unpaired, Student’s two-tailed t-test. *p < 0.05, **p < 0.005, ***p < 0.0005.

When lipid raft structures are disrupted, changing the localization of CD1d to include non-raft regions, NKT cell stimulation is radically attenuated^25^. We tested this important concept *in vitro* and *in vivo*. First, we pre-loaded peritoneal macrophages from WT versus *Abca7*^−/−^ mice with titrated doses of alpha-galactosylceramide (αGalCer), a strong activator of NKT cells, followed by co-culturing those cells with Vα14i NKT-cell line overnight. NKT cell activation was measured by IFNγ and IL-4 production via ELISA (Fig. 5A,B). We found diminished production of both cytokines from the NKT cells cultured with *Abca7*^−/−^ peritoneal macrophages. We repeated this assay with DP thymocytes isolated from WT and *Abca7*^−/−^ mice and found a similar defect in activation from those NKT cells cultured with the *Abca7*^−/−^ DP thymocytes (Fig. 5C,D). These data are consistent with a partially blocked maturation of NKT cells in these mice, rather than a preferential differentiation of NK1.1^−^ NKT2 cells^44^,^45^. Furthermore, they demonstrate a functional defect in the ability of ABCA7-deficient antigen presenting cells to properly activate NKT cells through CD1d. We verified this defect through an *in vivo* activation assay. We administered αGalCer to WT and *Abca7*^−/−^ mice and after 2 hours we analyzed NKT cell activation based on IL-4 and IFNγ production measured by intracellular staining and flow cytometry (Fig. 5E). We found that *Abca7*^−/−^ antigen presenting cells failed to efficiently activate NKT cells after αGalCer administration *in vivo*. Overall, these results demonstrate that ABCA7, in thymocytes and antigen-presenting cells, is important for CD1d surface expression and lipid raft content on the cell surface. The absence of ABCA7 results in the reduction of both factors, resulting in a failure of APC and thymocytes to efficiently activate NKT cells.

## Discussion

In this study, we demonstrate that changes in surface CD1d expression and intracellular trafficking, as well as reduced CD1d localization to lipid rafts, are all caused by the absence of ABCA7. Defects in CD1d expression and lipid raft content were functionally significant, as there was a reduction in NKT cell cytokine production in response to antigen stimulation *in vitro* and *in vivo*. Similar to APCs, *Abca7*^−/−^ DP thymocytes likewise displayed lower surface CD1d, overall a reduced lipid raft content, and reduced co-localization of CD1d in lipid rafts. As a likely consequence, NKT cell development also was impaired. Utilizing a mixed bone marrow chimera approach, we verified a cell-extrinsic mechanism, meaning that the defects observed in *Abca7*^−/−^ mice do not occur within the NKT cell precursor, but that more likely ABCA7 deficiency adversely affects the DP thymocyte population that is responsible for mediating NKT cell positive selection.

ABCA7-deficient thymic NKT cells displayed reduced proliferation *in vivo* and defective maturation through the early stages of development. Considering the number and frequency of thymic NKT cells, as well as their reduced proliferation, the block in development was particularly evident at or just after stage 1. There were no differences in numbers of apoptotic NKT cells in thymus. We anticipate that the reduced numbers of NKT cells at stages 1–2 were likely due, at least in part, to reduced proliferation. However, we cannot rule out that possibility that there was also some reduced differentiation of lymphocytes towards the NKT lineage in thymus.

Wang *et al*. found that ABCA7 has the ability to bind apolipoproteins and promote efflux of cellular phospholipids^3^. More recently, Quazi *et al*. reported that ABCA7 actively exports or phosphatidylserine from the cytoplasmic to the exocytoplasmic leaflet of membranes; therefore, ABCA7 acting to transport lipid molecules from intracellular compartments to the surface of cells has been established^9^. ABCA7 and CD1d appear to be colocalized within the same compartment (Supplemental Fig. 3A) and in the absence of ABCA7, CD1d is found localized at a higher frequency within late endosomal compartments (Fig. 3). However, we were unable to successfully perform co-immunoprecipitations of CD1d and ABCA7 due to technical issues, so we do not know that these two proteins directly bind to each other. ABCA7 may function within the endosome, assisting with the loading of processed lipid or phospholipid antigen onto CD1d for trafficking of the receptor complex out of late endosomal compartments to the cell surface. In this way, ABCA7 would be functioning analogously to the transporter for antigen processing (TAP), which pumps peptides into the endoplasmic reticulum for loading into MHC class I proteins^46^,^47^.

CD1d molecules localize to ‘steady-state’ membrane lipid rafts and this localization is crucial for the presentation of antigen. Very recent work by Torreno-Pina *et al*.^42^ has elegantly shown through novel super-resolution microscopy that human CD1d localizes in clusters on the plasma membrane of antigen-presenting cells (APCs). Further, the density of this clustering controls NKT cell activation by APCs. Based on our results, we believe that deficiency of ABCA7 alters lipid raft content and distribution, resulting in decreased co-localization of CD1d within lipid rafts. Furthermore, in the absence of ABCA7, surface CD1d aggregation or clustering within the lipid rafts is reduced. Previous studies have shown that activation of surface receptors in hematopoietic cells results in their enrichment or aggregation within lipid rafts^48^,^49^. As lipid rafts function as centers of signal transduction, aggregation of lipid rafts following receptor ligation is regarded as a general mechanism for promoting immune cell signaling. Our work is consistent with this notion that reduced clustering of CD1d controls NKT activation.

The results of this current study further illustrate how changes in intracellular phospholipid or cholesterol impact immune cell function *in vivo*. Several prior studies have shown how lipid accumulation in lymphocytes impacts their differentiation and activation^32^,^33^,^50^,^51^,^52^,^53^. Accumulation of intracellular cholesterol stimulates CD4^+^^32^,^33^ and γδ^53^ T cell proliferation and activation. Specifically, with regard to NKT cells, deficiency in either the ABC transporters *Abcg1, Abca7*, or in the Niemann-Pick Type C1 protein (*Npc1)* inhibit NKT cell development and function. However, they impact NKT development in different ways. Although ABCG1 affects lipid rafts through the regulation of cellular cholesterol content, *Abcg1* deficiency causes a cell-autonomous defect in the NKT cell precursor, as shown by our group^29^, and this is not related to an antigen presentation defect, as is the case for ABCA7. *Npc1* is involved in the mobilization of cholesterol within cellular compartments. *Npc1*-deficient mice have defects in lipid trafficking from the endosome to the lysosome^41^; however, to date, no evidence has been provided regarding the effect of *Npc1* on lipid rafts. *Abca7* deficiency results in a different effect, in that CD1d in the antigen-presenting cell is trapped within the late endosomal compartment, thus, preventing proper engagement of CD1d with the invariant TCR on the NKT cell. The role of another cholesterol transporter, ABCA1, in regulating NKT development has not been studied. All in all, these data illustrate that cholesterol and phospholipid transporters play significant roles in modulating NKT cell development and function. Further, these findings suggest that single nucleotide polymorphisms (SNPs) that functionally change expression of any one of these lipid transporters could have a significant impact on NKT cell function. Although associations of SNPs in these genes with NKT or lymphocyte function has not been studied, SNPs in ABCA7^54^, ABCG1^55^, NPC1^56^, and ABCA1^57^ have been associated with various lipid-based diseases, including Alzheimer’s, cardiovascular disease, obesity, Type 2 diabetes, and hypertriglyceridemia.

In summary, we demonstrate a novel role for ABCA7 in CD1d surface expression and antigen presentation function. As a consequence, absence of ABCA7 has a significant impact on NKT cell development and activation. NKT cells have been implicated in the development of atherosclerosis, autoimmunity, rheumatoid arthritis, and several forms of allergies. All of these diseases are in part due to “over-activation” of NKT cells. Therefore, linking ABCA7 with NKT cell activation could lead to the development of entirely new therapeutic approaches for these and other diseases.

## Materials and Methods

### Mice

C57BL/6 J wild-type mice (000664), B6.129S7-Rag1^tm1Mom^/J (002216) and B6.SJL-*Ptprc*^*a*^ *Pepc*^*b*^/BoyJ (002014) CD45.1 mice were from The Jackson Laboratory. *Abca7*^−/−^ mice were generated in the Fitzgerald laboratory^10^, and were backcrossed for 10 generations onto a congenic C57BL/6 J background in the Hedrick laboratory. Mice were fed a standard rodent chow diet and were housed in microisolator cages in a pathogen-free facility. All experimental protocols presented within this manuscript were approved by the La Jolla Institute for Allergy and Immunology Animal Care and Use Committee, and were performed according to criteria outlined in the Guide for the Care and Use of Laboratory Animals from the National Institutes of Health. Mice were euthanized by CO_2_ inhalation followed by cervical dislocation.

### Flow Cytometry and Antibodies

Thymus and lymph nodes were excised and pushed through a 70-μm strainer, and bone marrow cells from both femurs and tibias were collected by centrifugation. All samples were collected in Dulbecco’s PBS (Gibco) and were stored on ice during staining and analysis. Red blood cells were lysed in RBC Lysis Buffer according to the manufacturer’s protocol (BioLegend). Cells (2 × 10^6^ to 4 × 10^6^) were resuspended in 100 μl flow staining buffer (1% BSA (wt/vol) and 0.1% (wt/vol) sodium azide in PBS). Fcγ receptors were blocked for 15 min and surface antigens on cells were stained for 30 min at 4 °C. LIVE/DEAD Fixable Dead Cell Stain (Invitrogen) was used for analysis of viability, and forward- and side-scatter parameters were used for exclusion of doublets from analysis. For intracellular cytokine staining, cells were stimulated for 2 h with phorbol myristate acetate (50 ng/ml) and ionomycin (1 g/ml; Sigma-Aldrich) in the presence of brefeldin A (GolgiPlug; BD Biosciences). For additional intracellular staining, cells were fixed and made permeable with the Cytofix/Cytoperm Fixation/Permeabilization Solution Kit (BD Biosciences). Cells were stained for 30 min at 4 °C with directly conjugated fluorescent antibodies. The absolute number of cells was calculated by multiplication of the percentage of live cells in individual subsets by the total cell count before staining. Calculations of percentages were based on live cells as determined by forward and side scatter and viability analysis. Cell fluorescence was assessed with a FACSCalibur (BD Biosciences) and was analyzed with FlowJo software (version 9.2). Mean fluorescence intensity was quantified, and expression was calculated relative to that of the wild-type control. For staining of thymocytes from mice, thymi were collected and prepared as previously described and 5 × 10^6^ cells were incubated for 30 min at 4 °C in 30 μl flow staining buffer (1% (wt/vol) BSA and 0.1% (wt/vol) sodium azide in PBS) with the appropriate antibodies in the presence of Fc Block (BD Biosciences). Cellular fluorescence was assessed with an LSR II, FACSAria II or FACSCalibur (BD Biosciences) and data were analyzed with FlowJo software (TreeStar). Flow cytometry antibodies, including anti-mouse CD45.2 (104), CD4 (RM4-5), TCRβ (H57-597), IL-4 (BVD6-24G2), CD44 (IM7), NK1.1 (PK136), and CD1d (1B1), were purchased from eBioscience (San Diego, CA); CD45.1 (A20), IFN-γ (XMG1.2) were purchased from BD Biosciences (San Jose, CA); CD19 (6D5) and CD8α (5H10) were purchased from Invitrogen (Carlsbad, CA. Allophycocyanin-conjugated CD1d tetramers loaded with PBS-57 (an α-GalCer analog) were provided by the National Institutes of Health Tetramer Facility. Anti-CD3ε (145-2C11), anti-CD28 (37.51), and CD16/CD32 (2.4G2) antibodies were purchased from BD Biosciences.

### Immunoprecipitation Assay

Thymus from wild type and knock out mice were harvested and immediately lysed in 10ul/mg NP-40 lysis buffer with 1x protease inhibitor cocktail (ThermoFisher) and homogenized with a PowerGen125. Lysates were incubated at 4 **°**C on a rotor for 2 hours before spinning down at 12000 rpm for 20 min at 4 **°**C in a microcentrifuge. Total protein concentration was determined by BCA assay and 200ug of 1 mg/mL total protein lysate was used for co-immunoprecipitation. Half the starting lysate was loaded onto 50uL of crosslinked protein G Dynabeads (ThermoFisher) with rat anti mouse ABCA7 (LS-B222, LSBio) overnight at 4 **°**C on a rotor. Beads were separated from lysates with a magnetic stand, washed 3 × with 100uL cold PBS before adding 100uL1x LDS (ThermoFisher), with or without β-mercaptoethanol, and incubated at 70 **°**C for 10 minutes at 500 rpm in a Thermomixer (Eppendorf). Equal protein amounts of each fraction were loaded into a 4–12% Bis-Tris gel (ThermoFisher) and transferred onto a nitrocellulose membrane. Co-IP of either CD1d or MHCII was detected using 1:1000 rat anti mouse CD1d (clone 1B1, BD Biosciences) or rabbit anti mouse MHCII (clone ab180779, Abcam) and 1:5000 secondary goat anti rat IRDye 800CW or goat anti rabbit IRDye 680RD (Licor). The CD1d antibody clone 1B1 has been shown to recognize both mouse and rat CD1d^58^. Odyssey software was used to collect and analyze blots.

### Confocal microscopy

Resident murine peritoneal macrophages from WT or *Abca7*^−/−^ mice were obtained by lavage of peritoneum with PBS, and seeded onto glass cover slips pre-coated with fibronectin in 12-well tissue culture plates. Cells were fixed in 3% paraformaldehyde in PBS for 15 min at room temperature. Fixed cells were permeabilized using 0.2% Triton X-100 in PBS for 10 minutes. Then cells were incubated in blocking buffer (10% Fetal bovine serum in PBS) for 1 h prior to 45 minutes to 1-hour incubation with respective primary antibodies (diluted using blocking buffer) followed by washing (3 times) with PBS/T (0.5%Tween 20 in PBS) and a single wash with PBS. Cells were further incubated with secondary antibody and washed with PBS/T (0.5% Tween 20 inPBS) and incubated with NucBlue^®^ Fixed Cell Reagent (Thermofisher) in PBS for 5 minutes for nuclear staining followed by three washes with PBS. All cover slips were mounted on slides with antifade (Thermofisher).

Thymocytes were seeded onto poly-L lysine coated slides at 37 C for 2 hours. Cells were then fixed in 3% paraformaldehyde in PBS for 15 min at room temperature. Cells were incubated in blocking buffer (10% Fetal bovine serum in PBS) for 1 h prior to 45 minutes to 1-hour incubation with respective antibodies (diluted using blocking buffer) followed by washing (3 times) with PBS. Cells were then stained with NucBlue^®^ Fixed Cell Reagent (Thermofisher) in PBS for 5 minutes for nuclear staining followed by three washes with PBS. All cover slips were mounted on slides with antifade (Thermofisher).

Multi-labeled sample slides of samples were imaged with an Olympus FV10i Laser Scanning Confocal microscope (Olympus, Center Valley, PA). Using the FV10i acquisition software, each circular coverslip of cells was separated into 4 3 paneled mega-images. Each z series panel (1024 × 1024) was serially acquired with a 60 × objective using a mechanical step size of 0.3 microns between sections and then stitched together through a 10% overlap with the Olympus FluoView 1000 (Olympus) imaging software. Multi-labelled images were maximum projected and imported into Image Pro Premier (IPP) (Media Cybernetics, Inc MD) for further quantitative analysis, including colocalization assessment. Quantitative analysis of CD1D or Cholera Toxin or Caveolin1 or LAMP-1 staining intensity/dynamic range was obtained after thresholding and extracting true signals based on control samples. These thresholds of dynamic range were used to obtain Manders correlation coefficients of the various paired stainings using the IPP software colocalization module. An average of 3 experiments and 200 cells per condition are represented in figures.

### Lipid Raft Staining

Thymocytes or splenocytes were isolated from tissue by being pushed through a 70-μm strainer. All samples were collected in Dulbecco’s PBS (Gibco) and were stored on ice during staining and analysis. Red blood cells were lysed in RBC Lysis Buffer according to the manufacturer’s protocol (BioLegend). Cells (2–4 × 10^6^) were resuspended in 100 μl flow staining buffer (1% BSA (wt/vol) and 0.1% (wt/vol) sodium azide in PBS). Fcγ receptors were blocked for 15 min and surface antigens on cells were stained for 30 min at 4 °C. FITC-conjugated Cholera Toxin B (CTB) was used to stain lipid rafts. Samples were analyzed by flow cytometry.

### Mouse Vα14i NKT-cell line

Thymocytes from WT mice were enriched for Vα14i NKT cells by magnetic depletion using biotinylated antibodies against CD8α, CD19, CD24 and TER-119 (BD Biosciences and eBioscience) together with EasySep magnets and protocols and reagents from StemCell Technologies. Cells were then stained with αGalCer-loaded CD1d tetramers, together with anti-TCRβ antibodies, in staining buffer containing 1 μg/mL streptavidin. Tetramer-positive, TCRβ+ cells were isolated using a FACSAria cell sorter (BD Biosciences). Sorted Vα14i NKT thymocytes were then cultured for 48 hours at 10^6^/mL in complete RPMI media (supplemented with 10% FBS, 50 μM β-mercaptoethanol, 1X penicillin/streptomycin/glutamine mix, and 20 mM Hepes) on a plate coated with anti-TCRb antibody together with soluble anti-CD28. Cells were then maintained by culture in complete RPMI media with 10ng/mL mouse IL-15/IL-15Ra (eBioscience) for 5 days before being used in experiments.

### Antigen presentation assay

Resident peritoneal macrophages were isolated from WT and *Abca7*^−/−^ mice. Cells were washed and plated on to a 96 well plate. Macrophages were then incubated with αGalCer at concentrations indicated for 6hrs. Antigen was washed away, and macrophages were incubated with the mouse Vα14i NKT-cell line overnight. After 16 h, cell supernatants were collected and IFN-γ and IL-4 were measured by ELISA (eBioscience) according to manufacturer’s protocol.

### NKT activation assays

For activation of primary *i*NKT cells, 24-well plates were coated with 5 μg/ml αCD3ε antibody in PBS at 4 °C overnight. The next day, the plates were washed twice with PBS, and thymocytes were plated at 2 × 10^6 ^cells/well in RPMI 1640 medium supplemented with 5% FBS and 1% penicillin/streptomycin. Soluble αCD28 antibody (2 μg/ml) and GolgiPlug (BD Biosciences) were added, and the cells were incubated at 37 °C for 4 h. Thymocytes were stimulated with PMA (1 μg/ml) and ionomycin (200 ng/ml) for 4 h in the presence of GolgiPlug at 37 °C. IL-4 and IFN-γ production by *i*NKT cells was assessed by flow cytometry following methods described in our prior publications^59^,^60^.

*In vivo* BrdU proliferation assay and detection of apoptosis. C57BL/6 (WT) and *Abca7*^*−*/*−*^ mice were injected i.p. with 0.3 mg BrdU (in 100 μl PBS) three times every 4 h. Thymi were harvested the next day, and single-cell suspensions were stained with fluorophore-conjugated Abs and CD1d tetramer. After cell surface staining, cells were analyzed for BrdU incorporation using FITC or allophycocyanin BrdU flow kit (BD Biosciences), according to the manufacturer’s instructions. Apoptosis of thymic *i*NKT cells was measured by flow cytometry using a PE-Annexin V Apoptosis Detection Kit 1 (BD Biosciences), according to the manufacturer’s instructions.

Generation of bone marrow chimeras. Recipient mice (*Rag1*^*−*/*−*^) were irradiated in two doses of 450 rad each (for a total of 900 rad) 4 h apart. Bone marrow cells from both femurs and tibias of donor mice (B6.SJL and *Abca7*^*−*/*−*^) were collected under sterile conditions. Bones were centrifuged for the collection of marrow, and cells were washed, mixed at 1:1 ratio, and resuspended in PBS for injection. Approximately 5 × 10^6^ bone marrow cells from B6.SJL and *Abca7*^*−*/*−*^ mice (total 10^7^ cells) in 200 μl PBS were delivered retro-orbitally into each recipient mouse. Recipient mice were housed in a barrier facility under pathogen-free conditions before and after bone marrow transplantation. After bone marrow transplantation, mice were provided autoclaved acidified water with antibiotics (trimethoprim-sulfamethoxazole) and were fed autoclaved food. Mice were analyzed at 12 wk after bone marrow reconstitution.

### Delivery of αGalCer *in vivo*

αGalCer (Kyowa Hakko Kirin) was suspended in sterile saline. Mice were injected i.p. with either saline or 2 μg αGalCer as described^61^. At 2 h post-injection, livers were collected for NKT cell analysis.

## Additional Information

**How to cite this article**: Nowyhed, H. N. *et al*. ATP Binding Cassette Transporter ABCA7 Regulates NKT Cell Development and Function by Controlling CD1d Expression and Lipid Raft Content. *Sci. Rep.* **7**, 40273; doi: 10.1038/srep40273 (2017).

**Publisher's note:** Springer Nature remains neutral with regard to jurisdictional claims in published maps and institutional affiliations.

## Supplementary Material

Supplemental Figures
