# Supplementary material for: ATP Binding Cassette Transporter ABCA7 Regulates NKT Cell Development and Function by Controlling CD1d Expression and Lipid Raft Content
Source: Sci Rep. 2017 Jan 16;7:40273. doi: 10.1038/srep40273 (PMC5238393; doi:10.1038/srep40273)
Supplement: Supplemental Figures [file srep40273-s1.pdf]

Supplemental Information for:

ATP Binding Cassette Transporter ABCA7 Regulates NKT Cell Development and Function by Controlling CD1d Expression and Lipid Raft Content

Heba N. Nowyhed, Shilpi Chandra, William Kiosses, Paola Marcovecchio, Farah Andary, Meng Zhao, Michael L. Fitzgerald, Mitchell Kronenberg, and Catherine C. Hedrick

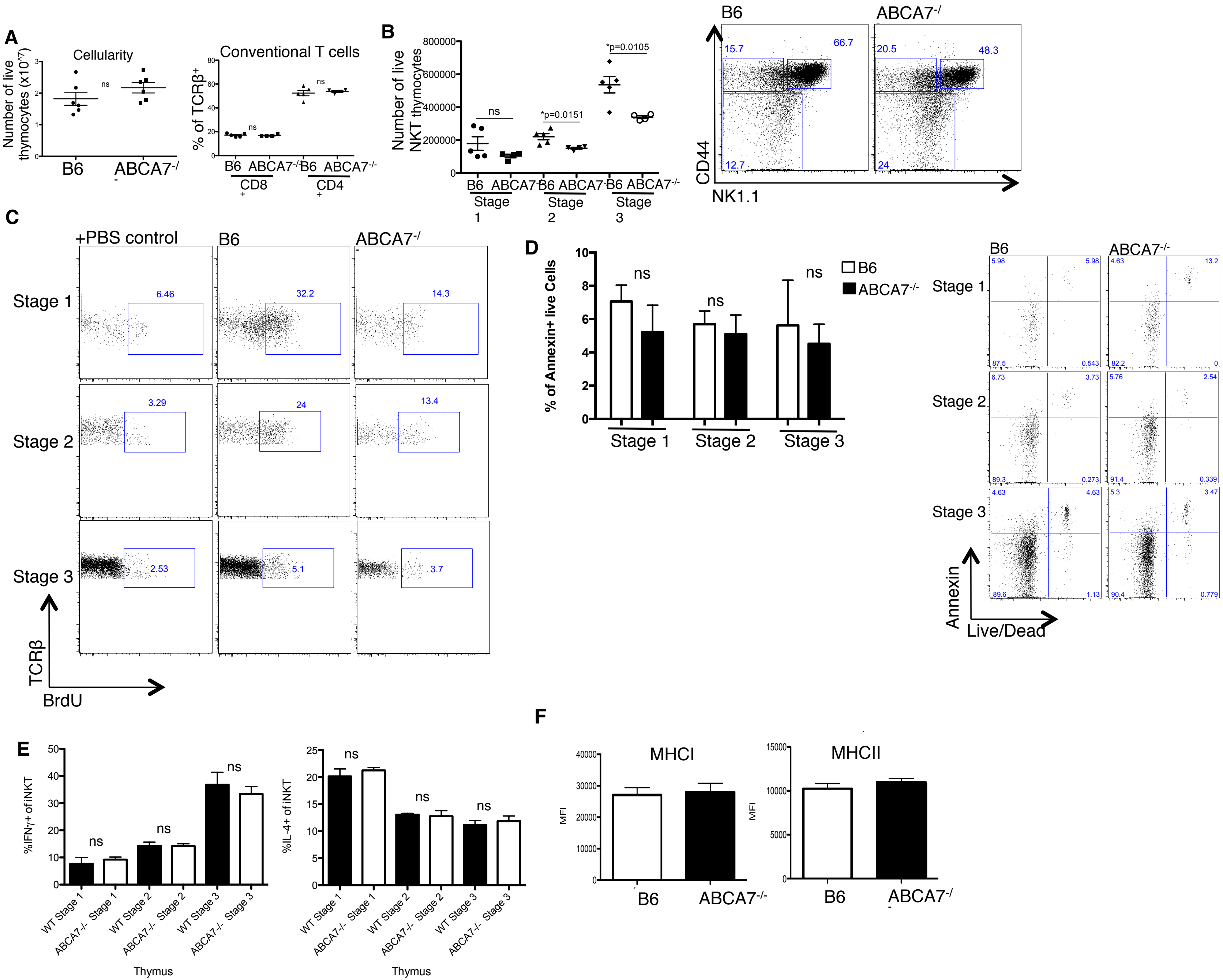

A
Cellularity
Number of live thymocytes (x10^7)
ns
B6
ABCA7-/-
Conventional T cells
% of TCRβ+
ns
B6 ABCA7-/- B6 ABCA7-/-
CD8+
CD4+
B
Number of live NKT thymocytes
ns
*p=0.0151
*p=0.0105
B6 ABCA7-/- B6 ABCA7-/- B6 ABCA7-/-
Stage 1
Stage 2
Stage 3
B6
ABCA7-/-
15.7
66.7
12.7
20.5
48.3
24
CD44
NK1.1
C
+PBS control
B6
ABCA7-/-
Stage 1
6.46
32.2
14.3
Stage 2
3.29
24
13.4
Stage 3
2.53
5.1
3.7
TCRβ
BrdU
D
% of Annexin+ live Cells
ns
ns
ns
B6
ABCA7-/-
Stage 1
Stage 2
Stage 3
B6
ABCA7-/-
Stage 1
5.98
5.98
87.5
0.543
4.63
13.2
82.2
0
Stage 2
6.73
3.73
89.3
0.273
5.76
2.54
91.4
0.339
Stage 3
4.63
4.63
89.6
1.13
5.3
3.47
90.4
0.779
Annexin
Live/Dead
E
%IFNγ+ of iNKT
ns
ns
ns
WT Stage 1
ABCA7-/- Stage 1
WT Stage 2
ABCA7-/- Stage 2
WT Stage 3
ABCA7-/- Stage 3
Thymus
%IL-4+ of iNKT
ns
ns
ns
WT Stage 1
ABCA7-/- Stage 1
WT Stage 2
ABCA7-/- Stage 2
WT Stage 3
ABCA7-/- Stage 3
Thymus
F
MHCI
MFI
B6
ABCA7-/-
MHCII
MFI
B6
ABCA7-/-

Supplemental Figure 1

A) Total number of live thymocytes and total number of live $TCRb^+CD4^+$ and $TCRb^+CD8^+$ T cells in the thymus of WT (B6) and *Abca7-/-* mice. B) Number of thymic NKT cell stages (Stage 1 $CD44^{lo}NK1.1^-$, Stage 2 $CD44^{hi}NK1.1^-$, Stage 3 $CD44^{hi}NK1.1^+$) in B6 versus *Abca7-/-* mice C) $BrdU^+$ NKT cells at Stage 1-3 of development D) Frequency of Annexin-V^+, Live cells of thymic NKT cell stages (Stage 1 $CD44^{lo}NK1.1^-$, Stage 2 $CD44^{hi}NK1.1^-$, Stage 3 $CD44^{hi}NK1.1^+$) in B6 versus *Abca7-/-* mice E) NKT cells from the thymus stimulated in vitro with anti-CD3 and anti-CD28 antibodies for 72 hours. Intracellular staining for IFNg and IL4 in NKT cells at Stage 1-3 of thymic development. F)MFI of MHCI and MHCII in thymocytes isolated from B6 and *Abca7-/-* mice. Data are representative of at least two separate experiments with at least three age and sex matched mice per group. P value, unpaired, Student's two-tailed t-test. *p<0.05. ns: not significant, p>0.05.

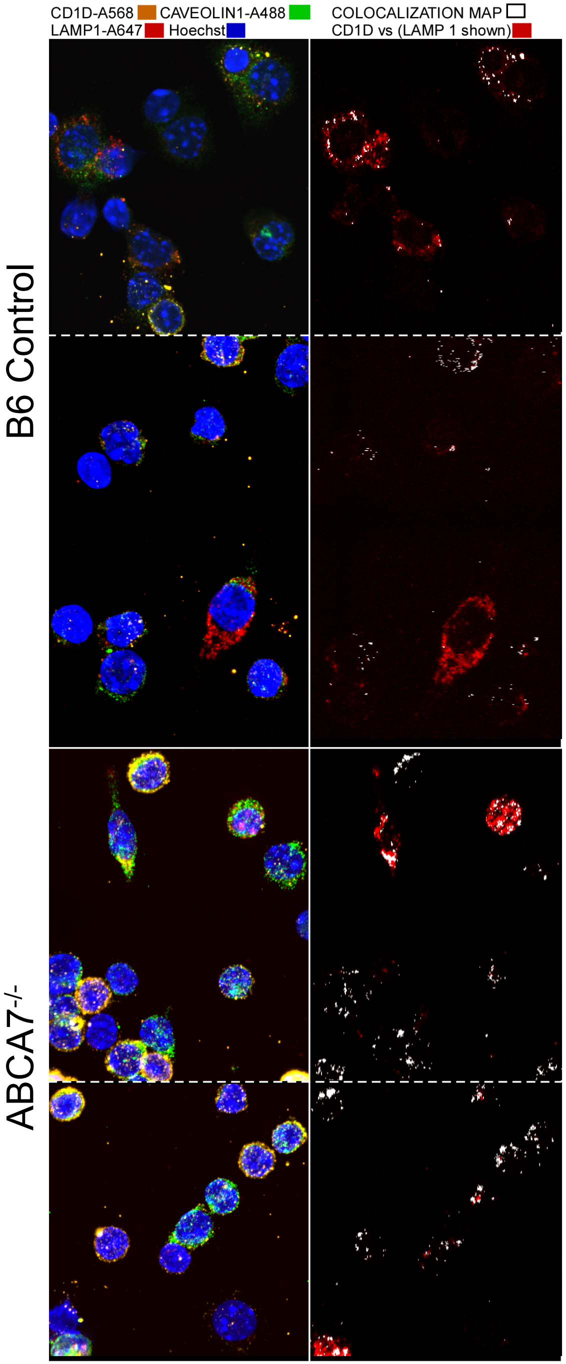

Supplemental Figure 2. Colocalization of CD1d and LAMP-1. Additional images showing colocalization by confocal microscopy of CD1d, Caveolin-1, and LAMP-1 on peritoneal macrophages harvested from WT and *Abca7*$^{-/-}$ mice (representative images). CD1d is shown in orange, caveolin in green, LAMP-1 in red, and Hoechst in blue. Left panels show all 4 markers; right panel shows colocalization of CD1d and LAMP-1.

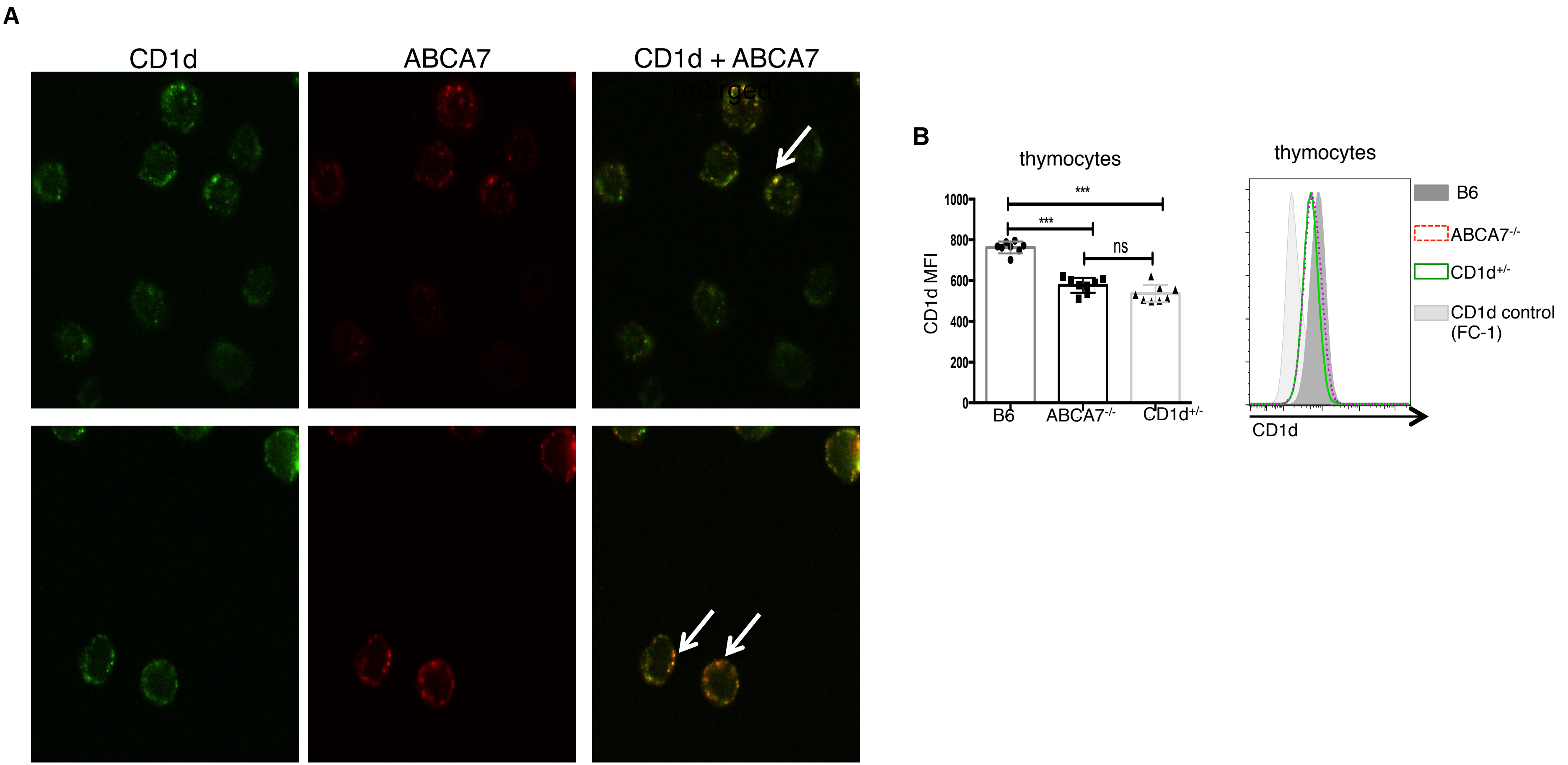

Supplemental Figure 3. A) Confocal microscopy of CD1d and ABCA7 co-localization on DP thymocytes isolated from WT (B6) mice. Top and bottom panels show two different images. White arrows point to some areas of colocalization. B) CD1d MFI on B6, *Abca7-/-*, and *CD1d+/-* thymocytes. Data are representative of at least two separate experiments with at least three age and sex matched mice per group. P value, unpaired, two-tailed t-test. ***p<0.0005. ns: not significant, p>0.05
